# Relationship Between the Periodontal Status of Pregnant Women and the Incidence and Severity of Pre-term and/or Low Birth Weight Deliveries: A Retrospective Observational Case-Control Study

**DOI:** 10.7759/cureus.31735

**Published:** 2022-11-21

**Authors:** Abhishek Sinha, Neha Singh, Anamika Gupta, Triveni Bhargava, Mahesh P. C., Pratiksha Kumar

**Affiliations:** 1 Department of Periodontology, Purvanchal Institute of Dental Sciences, Gorakhpur, IND; 2 Department of Periodontology, Dental Institute, Rajendra Institute of Medical Sciences (RIMS), Ranchi, IND; 3 Department of Public Health Dentistry, Yogita Dental College, Khed, IND; 4 Department of Oral and Maxillofacial Surgery, Government College of Dentistry, Indore, IND; 5 Department of Public Health Dentistry, Dayananda Sagar College of Dental Sciences, Bangalore, IND; 6 Department of Oral Pathology and Microbiology, Government College of Dentistry, Indore, IND

**Keywords:** case-control study, newborns, preterm birth weight, pregnant women, periodontal status

## Abstract

Background: Literature worldwide has linked periodontitis to preterm births and/or low-birth-weight infants. However, to our knowledge, research on this topic is scarce in India. The United Nations Children's Fund (UNICEF) reports that South Asian nations, particularly India, have the highest rates of preterm births and low-birth-weight infants as well as periodontitis due to poor socioeconomic conditions. Overall, 70% of perinatal fatalities result from prematurity and/or low birth weight, which also increases the incidence of morbidity and drives up the cost of postpartum care by a factor of ten. The frequency and severity of the illness may be greater among the Indian population due to their poor socioeconomic status. To lower the mortality rate and the cost of postnatal care, it is necessary to investigate the impact and severity of the effect of periodontal conditions on pregnancy outcomes in India.

Materials and methods: Following the collection of obstetric and prenatal records from the hospital in compliance with inclusion and exclusion criteria, a sample of 150 pregnant women were chosen from public healthcare clinics for the research. Within three days of delivery following enrolment in the trial, a single physician recorded each subject's periodontal condition using the University of North Carolina-15 (UNC-15) probe under artificial lighting and the Russell periodontal index. The gestational age was calculated based on the latest menstrual cycle, and a medical professional would order an ultrasound if they felt it was essential. The doctor weighed the newborns soon after delivery and in accordance with this prenatal record. The acquired data was analyzed using a suitable statistical analysis technique.

Results: The severity of a pregnant woman's periodontal disease was significantly correlated with the infant's birth weight and gestational age. Preterm births and low-birth-weight infants became more prevalent as the severity of the periodontal disease rose.

Conclusion: The results demonstrated that periodontal disease in pregnant women may increase the risk of preterm delivery and low birth weight in infants.

## Introduction

The concept that periodontal disease might influence systemic health is not new. Miller originally published his “focal infection theory” in 1981 suggesting that “microorganisms or their waste products gain an entry to parts of the body adjacent or remote from the mouth” [1]. Miller and other proponents of the focal infection concept have demonstrated connections between oral foci of infection and a wide variety of diseases, including tonsillitis, middle ear infection, pneumonia, tuberculosis, syphilis, osteomyelitis, endocarditis, meningitis, and septicaemia. Many teeth used to be removed prophylactically before the advent of current periodontal therapies; at that time, the focused infection idea was the guiding principle [2].

During an editorial in 1952, the Journal of the American Medical Association stated, “Many patients with disease caused by foci of infection have not been relieved of their symptoms by removal of the foci. Many patients with these same diseases have no focus on infection; also, foci of infection are as common in apparently healthy persons as those with disease” [3]. For the following 50 years, the focused infection notion was forgotten. Insights into the probable systemic routes of action of bacterial products and inflammatory cytokines, as well as recent breakthroughs in identifying and characterizing periodontal infections, have allowed for a more precise evaluation of the systemic impact of periodontal disease on systemic health. In this regard, a wide range of systemic health aspects may be impacted by periodontal inflammation and infection, from alterations in cardiovascular health and glycaemic management to chronic obstructive pulmonary disease (COPD) and even preterm low-birth-weight (PTLBW) deliveries.

Any live baby weighing less than 2500 g was designated as low birth weight (LBW) by WHO in 1995, while those weighing less than 1500 g were designated as very low birth weight (VLBW). WHO considers a birth that takes place before 37 weeks of pregnancy to be premature. PTLBW births are responsible for more than 33% of infant mortality, and even among the survivors, the prevalence of congenital neurological impairments and other developmental problems is elevated [4]. Initial hospitalization of these infants is also economically draining for the parents. Alcohol, smoking, drug use during pregnancy, advanced or advanced maternal age, low socioeconomic status, lack of prenatal care, low maternal BMI, hypertension, infections (especially genitourinary tract infections), cervical incompetence, diabetes, nutritional status, stress, and multiple pregnancies have all been linked to preterm birth (PB) and/or LBW. In excess of half of PB and/or LBW cases, none of these risk indicators is present. As a result, researchers have been seeking potential explanations, including the existence of persistent infectious disorders such as periodontal infection.

The emphasis on the uterus has turned away from other putative sources of infection due to the likelihood that diseases outside the fetal placental unit may impact preterm and low-birth-weight (PLBW) births. Periodontal disease has been related to an overgrowth of gram-negative bacteria in the periodontal tissues, leading to a delayed rise in local levels of prostaglandins and inflammatory cytokines and an increase in the number of synthetic flammables in the blood. PLBW births may be the result of periodontal disease in two ways: either through the production of inflammatory mediators or through a bacterial attack on the amnion. Notably, increasing attention is being given to the role that infection plays. Women who have asymptomatic bacteriuria or who are abnormally colonized by pathogenic micro-organisms are at an increased risk of having preterm births due to conditions including prelabour rupture of membranes, premature labour, and chorioamnionitis. Although the exact role of maternal infection in premature births is still up for debate, it is possible that elevated levels of inflammatory mediators, which shorten the gestational period, are a secondary result of an infection in the vaginal tract or elsewhere in the mother. There are several potential routes for microorganisms to acquire access to the choriodecidual sac and the amniotic fluid and foetus inside of it, including the vagina, the cervix, the endometrium (which may be chronically infected before pregnancy), and the bloodstream.

Periodontal disease, especially severe periodontitis, has been related to other systemic disorders, including chronic lung illness, asthma, insulin-dependent diabetes mellitus, and disorders of the heart and blood vessels (including endocarditis and coronary heart disease). One study found that PLBW babies were seven times more likely to be delivered by mothers with periodontal disease. Animal studies have also demonstrated that infection with microorganisms linked to gram-negative periodontitis might have an unfavourable impact on the mother and her unborn child. When the periodontal infection Porphyromonas gingivalis was injected subcutaneously into the dorsal region of pregnant hamsters, the birth weight of the offspring was reduced by 25% compared to offspring born to healthy mothers.

The current research intended to examine whether the prevalence of a periodontal infection was connected to PTLBW after controlling for other known obstetric risk factors and confounders. Periodontal infection is a known risk factor in PTLBW.

## Materials and methods

The study was conducted in the public health care clinic in the concerned area. Ethical clearance was obtained, and the IRB number was PIDS/ 2021/145. Before conducting the study, approval was obtained from the ethical committee. Prior to performing any examination, we made sure to acquire the patients’ approval for the treatment. Patients were included in a case-control study during the first 72 hours after giving birth. They were examined in accordance with a pre-formed questionnaire and periodontal examination charts. Afterwards, the patients were examined by a single examiner for the periodontal index given by Russell using the following clinical armamentarium.

Then, the mothers’ and infants’ birth weights, and any obstetric risks were double-checked against the patients’ hospital records. The following variables were recorded: name, age sex, address, and occupation, nourishment status, the presence of anaemia during the pregnancy, the presence of sexually transmitted diseases such as HIV and syphilis, history of tobacco usage and alcohol drinking during pregnancy, usage of antibiotics during the pregnancy, date of delivery, period of the gestation, the weight of the infant, and any postnatal complications. After recording all the variables, 100 patients were selected after satisfying all the inclusion and exclusion criteria. Inclusion criteria included the following: duration of fewer than three days from giving birth, a normal or above average BMI, and natural conception and healthy pregnancy and delivery. The participants ranged in age from 18 to 35 years old. The exclusion criteria included the following: pregnancy-related urinary tract infections or bacterial vaginosis, previous exposure to antibiotics during pregnancy, prenatal history of alcohol or cigarette use, family history of premature birth, LBW, PTLBW children, abortion, or stillbirth, history of hypothyroidism, hypertension or diabetes, and sexually transmitted diseases (HIV, syphilis, etc.).

Afterwards, the study population was divided into two groups: Group I (control) included infants born at 37 weeks or more with a birth weight of more than 2500 g, considering them to have been born to "term" (term normal weight infants, TNBW); Group II (case) was the larger group and included the following subsets:

Subgroup A (preterm normal weight, PNBW): This group included the mothers whose infants were born at less than 37 weeks of pregnancy and weighed more than or equal to 2500 g.

Subgroup B (term low birth weight, TLBW): This group included the mothers whose infants were born at 37 weeks or more and were considered to have been born at a healthy weight of less than 2500 g.

Subgroup C (preterm low birth weight, PTLBW): This group included the mothers who gave birth to infants under 2500 g at a gestational age of 37 weeks.

For statistical analysis, the periodontal index values of the experimental group were compared to those of the control group. We determined their means, medians, and standard deviations. The Mann-Whitney U test was used to ascertain whether there is a connection between the qualitative periodontal statuses and the outcomes of the pregnancies. Without including the periodontal score, a chi-square test was used for the univariate analysis to determine whether or not there was a correlation between the dependent (pregnancy) and independent (other factors) variables. To examine whether or not any of the variables were linked to the pregnancy outcome after controlling for confounding factors, a logistic regression analysis was conducted. It was carried out for the primiparous mothers for the main risk factors found in the analysis done for all subjects.

## Results

The total data collected were tabulated according to all the variables recorded. A total of 100 subjects were examined and categorized into two groups: 28 cases and 72 controls.

The cases were mothers (A) who delivered infants after 37 weeks of gestation with a birth weight below 2500 g, (B) who delivered infants before 37 weeks of gestation with a birth weight above 2500 g, and (C) who delivered infants before 37 weeks of gestation with a birth weight below 2500 g. The controls were the mothers who delivered infants after 37 weeks of gestation with a birth weight above 2500 g. The periodontal status for each mother was calculated according to the periodontal index given by Russell. To nullify the effect of previous pregnancies, the data for the primiparous mothers were pooled to form the groups of cases and controls separately.

Regarding the distribution of the study population according to the maternal demographic variables, Table 1 presents the demographic data for the sample population.

**Table 1:** Distribution of study respondents by different characteristics

Factors	Cases	Control	No of respondents
Age			
<25yrs	25	44	69
>25yrs	7	24	31
Teeth brushing habit			
Occasional	8	2	10
Regular	15	75	90
Prenatal examination visits			
<6	6	3	9
>6	20	71	91
Educational status			
Not graduated	9	5	14
Graduated	15	71	86
Household income			
<1lakh	4	4	8
>1lakh	25	67	92
Previous pregnancies			
0	10	49	59
1	16	25	41

The median age of the cases was 24.035, whereas the median age of the controls was 24.99. The ages of the cases and controls do not vary much. New-borns in the case group averaged 2.30 kilograms, whereas those in the average weight of the control group was 3.069 kilograms, indicating statistical significance. Furthermore, there was a statistically significant gap between the two groups in terms of age, with the cases having a much younger mean gestational age (36.61 weeks) than the controls (37.96 weeks) (Table2).

**Table 2:** Classification of newborn babies by weight of babies and period of gestation

Weight of baby	Period of gestation	Controls	Cases	Total
<2.5kg	<37weeks	0	7	7
	>37weeks	0	15	15
>2.5kg	<37weeks	0	6	6
	>37weeks	72	0	72
	Total	72	28	100

The mean BMI for the cases and controls was 21.69 kg /m2 and 22.78 kg/m2, respectively, indicating that this factor was not statistically significant. Regarding the difference in periodontal status between the cases and controls, the periodontal score was defined by the periodontal index score recorded for the cases and controls. When compared to the controls, whose mean score was 0.7206 and standard deviation was 0.3940, the prevalence of periodontal disease was higher in the cases (mean score: 2.3693, SD: 0.8772). Thus, a statistically significant link was established between a high periodontal index score and a higher risk of adverse birth outcomes using the Mann-Whitney U test (p< 0.05) (Table 3).

**Table 3:** Comparison of controls and cases with pi scores by Mann-Whitney U test *p<0.05 n- number; PNBW- Pre-term normal weight delivery; TLBW- Term low birth weight delivery; PTLBW- Pre-term low birth weight delivery

Groups	n	Mean	SD	Median	Sum of ranks	U-value	Z-value	P-value
Controls	72	0.7206	0.3940	0.64	2728.00	100.00	-6.9706	0.0000*
Cases	28 PNBW 6 TLBW 15 PTLBW 7	2.3693	0.8772	2.68	2322.00			

Furthermore, the results of the Mann-Whitney U test indicated that the periodontal index score of the cases and controls who were also primiparous mothers was significantly different (p 0.05). The average periodontal disease score for the cases was 2.4054 (SD = 0.9728), whereas the average score for the controls was 0.6817 (SD = 0.3797) (Table 4). 

**Table 4:** Comparison of controls and cases with pi scores in primiparous mothers by Mann-Whitney U test *p<0.05

Groups	n	Mean	SD	Median	Sum of ranks	U-value	Z-value	P-value
Controls	46	0.6817	0.3797	0.59	1122.00	41.00	-4.7183	0.0000*
Cases	13	2.4054	0.9728	2.78	648.00			

The univariate analysis was done for the variables other than the periodontal status (independent variables), to check their association with the dependent variable, which was the pregnancy outcomes. The risk factors which were found to be significantly associated with the adverse pregnancy outcomes or cases were 1) Irregular teeth brushing habits 2) Pre-natal examination visits 3) Non-graduate educational level 4) Household income (Table 5).

**Table 5:** Univariate analysis of different factors with controls and cases *: significant

Factors	Chi-square	P-value
Age		
<25yrs	3.1405	0.0764
>25yrs		
Teeth brushing habit		
Occasional	21.1861	0.0000*
Regular		
Prenatal examination visits		
<6	12.1558	0.0005*
>6		
Educational status		
Not graduated	20.6514	0.0000*
Graduated		
Household income		
<1lakh	5.1339	0.0235*
>1lakh		
Previous pregnancies		
0	2.6719	0.2629
>1		

Additionally, the participants’ ages (more than or equal to 25 years old) were not significantly linked to the incidence of a poor pregnancy outcome. Similarly, no discernible link was noted between the number of previous pregnancies (> 1 vs. 0) and the chance of a poor outcome for the current pregnancy (Table 5). The periodontal index score, along with the other characteristics, was revealed to be substantially linked with unfavourable pregnancy outcomes in both the Mann-Whitney U test and the univariate analysis, which underwent logistic regression analysis. Irregular teeth brushing habit, prenatal examination visits, non-graduate educational level, household income less than 1 lakh. According to the logistic regression analysis, after controlling for the other variables, the variables which were found to be independent risk factors for the adverse pregnancy outcomes were the following: periodontal index score, non-graduate educational status, and >6 prenatal examination visits. Among these, the periodontal score (P = 0.000) demonstrated a strong association with adverse pregnancy outcomes, and the least association was with prenatal examination visits (P = 0.0470).

To determine if the primary risk variables identified in the logistic regression analysis for the whole study population, including educational level and the periodontal score, are also present in the primiparous subgroup of the population, a logistic regression analysis was performed. The results indicated that these two factors were strongly associated with adverse pregnancy outcomes after controlling for other variables. Between the two factors, a stronger association was noted with the periodontal score (P = 0.001) for the primiparous mothers. 

## Discussion

Due to its prevalence as a contributor to infant mortality and morbidity, obstetricians have devoted considerable time and energy to discussing and learning more about PB. Babies who are delivered early and/or weigh less than they should are more likely to have serious health problems, including severe cognitive impairments, epilepsy, and pathologic heart abnormalities [4]. The leading causes of death in newborns include being born prematurely and having a birth weight of less than 1500 g. This shows that a baby's birth weight is a good indicator of his or her future health, growth, and development.

Premature and/or underweight babies have been related to several different factors. 70 Premature delivery is often linked to infectious factors according to several studies. Inflammatory mediators that result from genitourinary tract infections such as bacterial vaginosis have been considered a physiologically feasible explanation for premature labour and early membrane rupture. It was also postulated that distant infections might contribute to premature LBW by way of the systemic circulation of bacterial vesicles and lipopolysaccharide (LPS). Nonetheless, the underlying processes of the hypothesized connection remain unclear [4].

The collection of infectious illnesses known as periodontal disease is characterized by the inflammation of the gingival and periodontal tissues and the eventual loss of alveolar bone. Many different kinds of microorganisms, mostly gram-negative anaerobic and microaerophilic bacteria, live in the subgingival space and are responsible for both the initial development and the ongoing maintenance of the periodontal infection. The host's immune system plays a crucial role in the development of periodontal disease. Researchers have proposed that the pathogenesis of periodontal disease and PTLBW are similar to those of other maternal disorders [5]. Pro-inflammatory cytokines produced by inflamed periodontal tissues may have systemic effects. The cytokines interleukin-1, IL-6, prostaglandin E2, and tumour necrosis factor-alpha (TNF-α) are all good examples. 73 Birth weight in premature infants may be low due to the presence of periodontal disease, either by a direct bacterial attack on the amnion or an indirect process involving inflammatory mediators [6].

Evidence suggests that periodontal disease, particularly advanced periodontitis, is linked to a number of medical issues, including diseases of the heart and blood vessels (such as endocarditis and coronary artery disease), respiratory problems, and diabetes mellitus. In 1996, researchers Offenbacher et al. discovered that mothers with periodontal disease had a sevenfold increased risk of having a child with LBW. The periodontal infection Porphyromonas gingivalis was injected subcutaneously into the dorsal region of pregnant hamsters [7], and the ensuing offsprings were 25% lighter at birth compared to their healthy counterparts. Human case-control studies demonstrate that women whose babies were born early or had a low birth weight because they went into labour early or their membranes broke early are more likely to have advanced periodontal disease [2].

Using the results of the aforementioned literature review as a starting point, the current study sought to determine whether or not maternal periodontal disease is related to the nutritional condition of infants and therefore whether or not it is associated with LBW. To reduce selection bias in this study, the pregnancy outcome was recorded first, and then the periodontal stats of the cases and controls were accessed. Hence, it can be stated that the results were not influenced by selection bias. The periodontal statuses of the study population were recorded within 72 hours of pregnancy, ensuring that there was little change in the periodontal status of the pregnant women during this time. This case-control research accounted for potential confounding factors in a well-defined cohort, hence reducing the likelihood of selection bias (mothers giving birth at particular hospitals). When available, information from the maternity records was used to analyse the questionnaire's findings. Therefore, we have no reason to suspect that selection bias affected our findings.

In the present study, the mean age of the mother was 24.035 years (cases) and 24.99 years (control) in the complete data set. The cases were found to be younger than in Offenbacher dataset [8]. There was no significant difference between the cases and controls in terms of age, and this aligns with the findings of Davenport et al. [7] and Offenbacher et al. [8]. This result could be because the study population was aged between 18-35 years old and the risk of PTLBW increases below 18 years and above 35 years of age. Furthermore, the birth of infants with LBW has been linked to lower levels of parental education. This is because more women would seek out prenatal care if their level of education is higher. In this study, education was shown to significantly lower the risk of PLBW. The findings demonstrated that the risk of PLBW decreased as control subjects’ education levels rose. In the whole dataset, 11 cases and three controls did not graduate high school, and this difference was shown to be statistically significant in both the univariate and logistic regression analyses. This correlation was also seen in pregnancies with just one child. Additionally, Devenport et al. [7] found that higher levels of education among controls were correlated with lower rates of PLBW. On another note, this study ascertained that the frequency with which participants brushed their teeth was also significantly associated with a lower risk of developing PLBW. The results demonstrated that the risk of PLBW decreased when the controls maintained good oral hygiene.

Generally, research may employ many different ways to assess the severity of the clinical periodontal disease. In this study, the requirement to perform clinical examination on women who had just given birth motivated us to choose a periodontal index that could be used in a hospital setting. It was decided that Russell's periodontal index was the best screening tool because it is an epidemiologic index and has a real biological gradient in the environment it is meant for [9].

Comparing the average BMI of the healthy controls to that of the cases showed no statistically significant difference, and this finding aligns with Romero et al. [10], who likewise observed a broad range of BMI values (from 19.8 to 26.0 kg/m2) and found no statistically significant difference when comparing BMI to periodontal health. On the other hand, Buduneli et al. [11] demonstrated that mothers whose infants were born at an LBW gained much less weight than those whose infants were born at a healthy weight. Furthermore, this research noted a positive correlation between the frequency of prenatal visits and the health of the mother and her baby when treatment began early in pregnancy. Subjects with fewer than six prenatal visits were at greater risk for PLBW births. The number of such patients was marginally higher in some cases. Our results were similar to those of Offenbacher et al. [8]; however, Lopez et al. [12] and Dasanayake [13] did not find such an association.

Statistical analysis of the periodontal index scores revealed a significant difference between the groups (controls and cases), and this finding is consistent with previous work by Mokeem et al. [14]. Women who gave birth to infants with LBW had a higher risk of periodontal disease than women who gave birth to infants with healthy weight at term. This is because periodontal bacteria may influence the outcome of pregnancy in both direct and indirect ways. Fetal damage from gram-negative periodontal bacteria has been hypothesized by Collins et al. in 1994 [15], but it depends on the severity of the infection. Reports have suggested that LPS from oral bacteria can cause adverse pregnancy outcomes, and studies have shown that LPS from Escherichia coli and Porphyromonas gingivalis can have a dose-dependent effect on fetal weight, with higher doses causing significant weight loss in the developing baby. According to a study published in 1998 by Dasanayake [13], LBW problems in infants have been linked to mothers' periodontal health. Periodontal disease is caused by gram-negative anaerobic bacteria, and the author speculates that these microorganisms may have an impact on the mother and baby. On the other hand, Davenport et al. [7] reported no connection between maternal periodontal disease and the likelihood of having a preterm or LBW infant. Actually, they discovered evidence that a higher mean pocket depth at delivery was linked to a lower risk of premature LBW. This inconsistency in results may be due to the fact that some research failed to appropriately account for potentially important confounding variables. Periodontal infections may have direct or indirect effects on the outcome of a pregnancy. Periodontal disease, which causes gum infections, is caused by gram-negative, anaerobic bacteria. Although genitourinary infections have been linked to adverse pregnancy outcomes, it is unclear whether or not certain periodontal diseases are associated with the fetoplacental unit. The prevalence of bacterial vaginosis and other genitourinary system illnesses in pregnant women has been associated with the delivery of babies with LBW. The gram-negative bacterium Bacteroides has also been linked to poor birth outcomes when it colonizes the vagina and cervix. Possible ways in which periodontal disorders might harm a growing fetus include inflammation of the placental membranes, which can occur even in the absence of an obvious infection. The first appeared in Offenbacher et al. in 1996 [8]. It has been theorized that gram-negative anaerobic bacteria in the periodontium may cause damage to the growing fetus via the production of endotoxins and maternal inflammatory mediators. The findings of the human studies are supported by those of the experimental studies conducted on pregnant hamsters, demonstrating that both periodontal disease and subcutaneous infection with a periodontal pathogen impair fetal development and increase levels of inflammatory mediators. LPS may be stored in the body due to persistent periodontal infection and then transported to the placental membranes through the bloodstream. Studies have revealed that LPS causes lL-1 and prostaglandin E2 (PGE2) to be produced by the chorioamniotic and trophoblastic cells, which is a common event before premature birth. On the other hand, periodontium may be a possible systemic source of fetotoxic cytokines owing to the production of inflammatory mediators such as PGE2 and TNF-alpha there. Additionally, new research has linked elevated blood TNF levels to the severity of disease progression in individuals with periodontitis who are experiencing active attachment loss [13,16].

There may also be an underlying problem, either genetic or environmental, that increases the likelihood of both periodontal disease and premature delivery. Both severe instances of periodontitis and LBW have been connected to the existence of a pre-existing hyper-responsive inflammatory characteristic. Multiple studies have linked periodontal infections to LBW and premature delivery due to a condition called subclinical bacterial vaginosis [17]. Therefore, further in-depth research is required. Since periodontal problems can be prevented and treated, these findings give new hope for trying to help people early in life to reduce the number of infants born too soon with too little weight.

The study's limitations were that it targeted a local population and had a small sample size.

## Conclusions

Multiple factors contribute to PB and LBW, making it a severe neonatal condition that causes hardship for families and a financial strain on the healthcare system. Smoking, heredity, alcohol intake, lack of prenatal care, insufficient maternal nutrition, urinogenital infection, and sexually transmitted diseases are all considered traditional risk factors. Premature birth and LBW are still poorly understood despite ongoing efforts to identify their root causes in the obstetrical context. Concerns have been raised about the link between maternal periodontal infection and unfavourable pregnancy outcomes, which is a growing topic of research due to the prevalence of premature and underweight infants. For over a decade, researchers have sought proof that periodontal disease and pregnancy are connected, but they have turned up with nothing. Thus, it is still a fascinating and attractive field to investigate, both in periodontology and obstetrics.
